# Supplementary material for: Machine learning for predicting the diagnosis of tuberculous versus malignant pleural effusion: External validation and accuracy in two different settings
Source: PLoS One. 2025 Sep 5;20(9):e0329668. doi: 10.1371/journal.pone.0329668 (PMC12412920; doi:10.1371/journal.pone.0329668)
Supplement: S1 Table — (DOCX) [file pone.0329668.s001.docx]

Supplementary Table 1: Number of cases by diagnosis obtained in the Training and Testing groups.

| Diagnosis | | | BAJO DEBA 1996-2012 (Testing group) | GIPUZKOA 2013-2022 (Training group) |
| --- | --- | --- | --- | --- |
| Malignant cases | | | 92 | 143 |
|  | pulmonary malignancy | | 48 | 88 |
|  | non-pulmonary malignancy | | 37 | 33 |
|  |  | unknown origin | 7 | 8 |
|  |  | urological origin | 5 | 2 |
|  |  | gastrointestinal origin | 6 | 6 |
|  |  | breast origin | 7 | 0 |
|  |  | gynaecological origin | 9 | 4 |
|  |  | throat origin | 3 | 0 |
|  |  | seminoma | 0 | 1 |
|  |  | sarcoma | 0 | 2 |
|  | lymphoma | | 5 | 13 |
|  | mesothelioma | | 2 | 9 |
| Other cases | | | 164 | 81 |
|  | heart failure and diuretic treatment | | 25 | 1 |
|  | parapneumonic after antibiotic treatment | | 46 | 27 |
|  | systemic disease | | 20 | 10 |
|  | haemothorax/post-traumatic | | 7 | 5 |
|  | chylothorax | | 3 | 2 |
|  | hormonal hyperstimulation | | 1 | 1 |
|  | post-thoracic surgery | | 6 | 6 |
|  | post-abdominal surgery | | 3 | 2 |
|  | haematological (non-lymphoma) | | 9 | 3 |
|  | hepatic hydrothorax | | 2 | 0 |
|  | pulmonary embolism | | 2 | 5 |
|  | hydrothorax/hydropneumothorax | | 4 | 1 |
|  | kidney disease | | 1 | 1 |
|  | hypothyroidism | | 1 | 0 |
|  | asbestosis | | 1 | 0 |
|  | no diagnosis | | 33 | 0 |
