# Supplementary material for: Machine learning for predicting the diagnosis of tuberculous versus malignant pleural effusion: External validation and accuracy in two different settings
Source: PLoS One. 2025 Sep 5;20(9):e0329668. doi: 10.1371/journal.pone.0329668 (PMC12412920; doi:10.1371/journal.pone.0329668)
Supplement: S2 Table — (DOCX) [file pone.0329668.s002.docx]

Supplementary Table 2: Diagnostic criteria met by tuberculous and malignant cases (listing each case just once under the highest quality criterion among all the criteria met).

| Diagnostic criteria | BAJO DEBA 1996-2012 (Testing group) | GIPUZKOA 2013-2022 (Training group) |
| --- | --- | --- |
| Positive Lowenstein culture | 21 | 26 |
| Granuloma in pleural tissue | 59 | 18 |
| ADA > 40 U/l in pleural fluid and complete recovery with specific treatment | 24 | 0 |
| Xpert MTB/RIF positive on pleural fluid | 0 | 5 |
| Malignant cells in pleural tissue | 28 | 133 |
| Positive pleural fluid cytology | 57 | 10 |
| Paramalignant | 7 | 0 |
