# Supplementary material for: Machine learning for predicting the diagnosis of tuberculous versus malignant pleural effusion: External validation and accuracy in two different settings
Source: PLoS One. 2025 Sep 5;20(9):e0329668. doi: 10.1371/journal.pone.0329668 (PMC12412920; doi:10.1371/journal.pone.0329668)
Supplement: Attachment — Submitted filename: Peer Review-Manuscript PONE-D-25-09667.pdf [file pone.0329668.s003.pdf]

Peer Review for Manuscript PONE-D-25-09667

Date: 9-April-2025

"Machine learning for predicting the diagnosis of pleural tuberculosis: external validation and accuracy in two different settings"

These are my comments of the peer review for the manuscript requested.

My general comments:

In general, the authors presented a well-conducted of external validation study. This research was developed, based on a authors' recent publication of development prospective cohort model (reported in 2021) of Adenosine deaminase (ADA) for pleural tuberculosis in low tuberculosis (TB) prevalence. The training cohort showed promising results, providing a good rationale for an external validation study. The analysis is very good; however, I think there is room for improvement in writing. To my own perception, I think the structure and presentation of this article writing are still not smooth.

The plus point of this external validation is that the developed pleural TB model (TPE) was tested in a completely different cohort with different TB/TPE incidence. In this study, although test cohort showed a bit lower accuracy, predictive values, than the Train study cohort. I think these real-life data are highly appreciated, demonstrating the applicability of the machine learning TPE models. This highlights the real-life data testing in the external validation cohort.

In clinical practice, tuberculous pleural effusion (TPE) is difficult for diagnosis, particularly in immunocompromised patients and/or limited resource countries with high TB burden. Hence, I raise a clinical question regarding the immune status of the study participants (training and test cohorts). Because HIV co-infection prevalence is highly significant among patients with TPE, do authors have any data about HIV infection or immune testing (CD4 cell counts…etc) in these cohorts?

In additionally, a short justification for sample size calculation in external validation cohorts enhances the validity of the study as well.

My specific comments:

1. **Title:** The title fully describes study aim and objectives.
2. **Abstract:** The abstract is well written. However, I also think one minor point for amendment, as below:

Lines 45 to 47:

The authors presented a sample size of the Test cohort with 832 consecutive patients in Bajo Deba health district (1996-2012), but did not show the sample size of the Train cohort from a prospective cohort study (2013-2020). To be consistent and transparent in data, the sample size (how many patients in Train cohort?) should be described (stated) in this section as well.

3. **Introduction:**

There is room for improvement in the introduction section, as follows:

Line 67: 95% UI needs to be fully written, as readers are not familiar with this term-UI.

Lines 92 to 94: "The model is freely 92 available as an app (at https://pleurapp.ispana.es/) to help other physicians or thoracic surgeons apply this approach when dealing with exudative and lymphocytic pleural effusions"

⇨ This information should not be placed in the introduction because it was not connected with the flow of the main idea discussed. This can be relocated to the discussion as appropriate.

Lines 99 to 100 in the introduction section:

"we compared the 99 diagnostic accuracy of the ML procedure and the classical Bayesian analysis system for TPE in both 100 different clinical scenarios **(Bajo Deba 1996-2012 and Gipuzkoa 2013-2022**)".

⇨ The authors mentioned two study cohorts to be modelled without brief introduction before. Hence, I recommend a brief introduction (1-2 sentences) of these 2 cohorts in the previous paragraphs.

4. **Materials and Methods and Results**

There is room for Materials and Methods section for improvement, regarding data presentation.

4.1 I can understand that the authors aimed to place emphasis on the Test cohort (external validation), so proactively present the Test study cohort as the first group, while the Train study cohort as the second group. Intuitively, this style of data presentation brings readers (like me) to

a certain level of confusion and needs to reread and rethink. Therefore, I recommend that the authors present data as routine to characterize **the first group = Train cohort, and second group = external validation cohort.**

This order of presentation should be consistent throughout the manuscript text and Tables.

In the **Tables 1 and 2**, the authors first present: *Test to Train cohorts (in sequence)*;

then in Tables 3 and 4: *Train to Test cohorts* (in sequence)

⇨ The transition in data presentation will make readers (eg, like my case) confused and take some time to reread, rethink the study data.

Therefore, I highly recommend a consistent presentation style of study data, I prefer training to testing cohort data presentation (from left to right), as the conventional way.

4.2 **In Lines 213 to 218**, the authors do not need to describe detailed data about confusion matrices of all machine learning models one by one, because readers can track all these information **in Figures 2 and 4** presented. Only salient features from these data should be stated in the manuscript text.

4.3 **Lines 237-239 and Lines 242-245**

"The estimated PPV area is calculated as a function of the pre-test probability (prevalence) using the sensitivity and specificity of each classifier obtained in the training dataset (Gipuzkoa):
(sensitivity * prevalence) / ((sensitivity * prevalence) + (1 - specificity) * (1 - prevalence))."

"The real PPVs in Gipuzkoa and Bajo Deba are calculated as the true positives divided by the sum of the true positives and false positives, obtained in each dataset: TP / (TP + FP)"

⇨ The formulas should be relocated in the Methods section, as it is more appropriate.

5. **Discussion:**

The discussion is comprehensively discussed and well written. From this study, the machine learning models outperformed the Bayesian modelling, as shown in a different study setting with different prevalence of TPE and malignancy.

Conclusion:

⇨ I think this is a great study, and minor amendments are suggested to make it more comprehensible for readers. I agree that this study is appropriate for publication.

Many thanks,

Best regards,
