# Supplementary material for: Machine learning for predicting the diagnosis of tuberculous versus malignant pleural effusion: External validation and accuracy in two different settings
Source: PLoS One. 2025 Sep 5;20(9):e0329668. doi: 10.1371/journal.pone.0329668 (PMC12412920; doi:10.1371/journal.pone.0329668)
Supplement: Attachment — Submitted filename: PONE-D-25-09667_reviewer.docx [file pone.0329668.s004.docx]

**PLOS ONE**

**Machine learning for predicting the diagnosis of pleural tuberculosis: external validation and accuracy in two different settings.**

## --Manuscript Draft--

| **Manuscript Number:** | PONE-D-25-09667 |
| --- | --- |
| **Article Type:** | Research Article |
| **Full Title:** | Machine learning for predicting the diagnosis of pleural tuberculosis: external validation and accuracy in two different settings. |
| **Short Title:** | Machine Learning for the predictive diagnosis in different clinical settings. |
| **Corresponding Author:** | Alberto Garcia-Zamalloa  Osakidetza Servicio Vasco de Salud Mendaro, Gipuzkoa SPAIN |
| **Keywords:** | pleural tuberculosis; Machine Learning; adenosine deaminase. |
| **Abstract:** | Objective.  To perform an external validation of a previously reported machine learning (ML) approach for predicting the diagnosis of pleural tuberculosis.  Patients and Methods.  We defined two cohorts: a Training group, comprising 273 effusions from our prospective study (2013-2020); and a Testing group, from a retrospective analysis of 360 exudative and lymphocytic pleural effusions from 832 consecutive patients in Bajo Deba health district (1996-2012). In training and test groups respectively, 49 and 104 cases were tuberculous, 143 and 92 were malignant, and 81 and 164 were diagnosed with “other diseases”; pre-test probabilities of pleural tuberculosis were 4% and 12.7%. Variables included were: age, pH, adenosine deaminase, glucose, protein, and lactate dehydrogenase levels, and white cell counts (total and differential) in pleural fluid. We used two ML classifiers: binary (tuberculous and non-tuberculous), and three-class (tuberculous, malignant, and others); and compared them with Bayesian analysis.  Results.  The best binary classifier yielded a sensitivity of 88%, specificity of 98%, and accuracy of 95%. The best three-class classifier achieved the same accuracy and correctly classified 83% (77/92) of malignant cases. The ML models yielded higher positive predictive values than Bayesian analysis based on ADA > 40 U/l and lymphocyte percentage ≥ 50% (92%).  Conclusions.  This external validation confirms the good performance of the previously reported ML approach for predicting the diagnosis of pleural tuberculosis based on exudative and lymphocytic pleural effusions, and for discriminating the cases most likely to be malignant. Additionally, ML was more accurate than the Bayesian approach in our study. |
| **Order of Authors:** | Alberto Garcia-Zamalloa |
|  | Rafael Arnay |
|  | Ivan Castilla-Rodriguez |
|  | Javier Mar |
|  | Jose Manuel Gonzalez-Cava |
|  | Oliver Ibarrondo |
|  | Iñaki Salegui |
|  | Juan Antonio De Miguel |
|  | Nekane Mugica |
|  | Borja Aguinagalde |
|  | Jon Zabaleta |
|  |  |

|  | Begona Basauri |
| --- | --- |
|  | Marta Alonso |
|  | Nekane Azcue |
|  | Eva Gil |
|  | Irati Garmendia |
|  | Jorge Taboada |
| **Additional Information:** |  |
| **Question** | **Response** |
| **Financial Disclosure**  Enter a financial disclosure statement that describes the sources of funding for the work included in this submission. Review the [submission guidelines](http://journals.plos.org/plosone/s/submission-guidelines#loc-financial-disclosure-statement) for detailed requirements. View published research articles from [*PLOS ONE*](https://journals.plos.org/plosone/) for specific examples.  This statement is required for submission and **will appear in the published article** if the submission is accepted. Please make sure it is accurate. | The author(s) received no specific funding for this work. |
| **Funded studies**  Enter a statement with the following details:   - Initials of the authors who received each award - Grant numbers awarded to each author - The full name of each funder - URL of each funder website - Did the sponsors or funders play any role in the study design, data collection and analysis, decision to publish, or preparation of the manuscript? |  |
| Did you receive funding for this work? |  |
| **Competing Interests**  Use the instructions below to enter a competing interest statement for this submission. On behalf of all authors, disclose any [competing interests](http://journals.plos.org/plosone/s/competing-interests) that could be perceived to bias this work—acknowledging all financial support and any other relevant financial or non- financial competing interests. | The authors have declared that no competing interest exist. |

| This statement is required for submission and **will appear in the published article** if the submission is accepted. Please make sure it is accurate and that any funding sources listed in your Funding Information later in the submission form are also declared in your Financial Disclosure statement.  View published research articles from  [*PLOS ONE*](http://journals.plos.org/plosone/) for specific examples. |  |
| --- | --- |
| **NO authors have competing interests**  Enter: *The authors have declared that no competing interests exist.*  **Authors with competing interests**  Enter competing interest details beginning with this statement:  *I have read the journal's policy and the authors of this manuscript have the following competing interests: [insert competing interests here]* |  |
| * typeset |  |
| **Ethics Statement**  Enter an ethics statement for this submission. This statement is required if the study involved:   - Human participants - Human specimens or tissue - Vertebrate animals or cephalopods - Vertebrate embryos or tissues - Field research   Write "N/A" if the submission does not require an ethics statement.  General guidance is provided below. Consult the [submission guidelines](http://journals.plos.org/plosone/s/submission-guidelines#loc-guidelines-for-specific-study-types) for detailed instructions. **Make sure that all** | N/A |

| **information entered here is included in the Methods section of the manuscript.** |  |
| --- | --- |
| **Format for specific study types**  **Human Subject Research (involving human participants and/or tissue)**   - Give the name of the institutional review board or ethics committee that approved the study - Include the approval number and/or a statement indicating approval of this research - Indicate the form of consent obtained (written/oral) or the reason that consent was not obtained (e.g. the data were analyzed anonymously)   **Animal Research (involving vertebrate animals, embryos or tissues)**   - Provide the name of the Institutional Animal Care and Use Committee (IACUC) or other relevant ethics board that reviewed the study protocol, and indicate whether they approved this research or granted a formal waiver of ethical approval - Include an approval number if one was obtained - If the study involved *non-human primates*, add *additional details* about animal welfare and steps taken to ameliorate suffering - If anesthesia, euthanasia, or any kind of animal sacrifice is part of the study, include briefly which substances and/or methods were applied   **Field Research**  Include the following details if this study involves the collection of plant, animal, or other materials from a natural setting:   - Field permit number - Name of the institution or relevant body that granted permission |  |
|  |  |
| **Data Availability**  Authors are required to make all data underlying the findings described fully available, without restriction, and from the time of publication. PLOS allows rare exceptions to address legal and ethical | Yes - all data are fully available without restriction |

| concerns. See the [PLOS Data Policy](http://journals.plos.org/plosone/s/data-availability) and [FAQ](http://journals.plos.org/plosone/s/data-availability#loc-faqs-for-data-policy) for detailed information.  A Data Availability Statement describing where the data can be found is required at submission. Your answers to this question constitute the Data Availability Statement and **will be published in the article**, if accepted. |  |
| --- | --- |
| **Important:** Stating ‘data available on request from the author’ is not sufficient. If your data are only available upon request, select ‘No’ for the first question and explain your exceptional situation in the text box. |  |
| Do the authors confirm that all data underlying the findings described in their manuscript are fully available without restriction? |  |
| **Describe where the data may be found in full sentences. If you are copying our sample text, replace any instances of XXX with the appropriate details.**   - If the data are **held or will be held in a public repository**, include URLs, accession numbers or DOIs. If this information will only be available after acceptance, indicate this by ticking the box below. For example: *All XXX files are available from the XXX database (accession number(s) XXX, XXX.*). - If the data are all contained **within the manuscript and/or Supporting Information files**, enter the following: *All relevant data are within the manuscript and its Supporting Information files.* - If neither of these applies but you are able to provide **details of access elsewhere**, with or without limitations, please do so. For example:   *Data cannot be shared publicly because of [XXX]. Data are available from the XXX Institutional Data Access / Ethics Committee (contact via XXX) for researchers who meet the criteria for access to confidential data.* | All the data are included in a database in our Department, and they cannot be shared publicly, but we have no problem to share all them for everyone who ask for it and who meet the criteria for access to confidential data. |

| *The data underlying the results presented in the study are available from (include the name of the third party and contact information or URL).*   - This text is appropriate if the data are owned by a third party and authors do not have permission to share the data.   * typeset |  |
| --- | --- |
| Additional data availability information: | Tick here if the URLs/accession numbers/DOIs will be available only after acceptance of the manuscript for publication so that we can ensure their inclusion before publication. |

<https://www2.cloud.editorialmanager.com/pone/download.aspx?id=39790606&guid=2cc7dffd-b72b-4bca-bf65-0844e2de48c4&scheme=1>Manuscript	[Click here to](https://www2.cloud.editorialmanager.com/pone/download.aspx?id=39790606&guid=2cc7dffd-b72b-4bca-bf65-0844e2de48c4&scheme=1)

[access/download;Manuscript;20250323_EXTERNAL_VALIDATI](https://www2.cloud.editorialmanager.com/pone/download.aspx?id=39790606&guid=2cc7dffd-b72b-4bca-bf65-0844e2de48c4&scheme=1)

1	Machine learning for predicting the diagnosis of pleural

2	tuberculosis: external validation and accuracy in two

3	different settings.

4 Garcia-Zamalloa A^1^., Arnay R^2^, Castilla-Rodriguez I^2^, Mar J^3^, Gonzalez-Cava JM^2^, Ibarrondo O^4^,
5 Salegui I^5^, De Miguel JA^5^, Mugica N^5^, Aguinagalde B^6^, Zabaleta J^6^, Basauri B^7^, Alonso M^8^, Azcue N^8^,
6 Gil E^9^, Garmendia I^10^, Taboada J^4^.
7 1.	Respiratory and Pleural Diseases Group. Biogipuzkoa Health Research Institute. Internal Medicine Service,
8 Osakidetza/Basque Health Service, Mendaro Hospital, Spain.
9 2.	Departamento de Ingeniería Informática y de Sistemas, Universidad de La Laguna, Santa Cruz de Tenerife, Spain.
10 3.	Epidemiology and Public Health Area, Economic Evaluation of Chronic Diseases Research Group, Biogipuzkoa
11 Health Research Institute, Donostia, Spain
12 4.	Western Gipuzkoa Clinical Research Unit, Osakidetza/Basque Health Service, Mendaro Hospital, Gipuzkoa, Spain.
13 5.	Respiratory and Pleural Diseases Group. Biogipuzkoa Health Research Institute. Pneumology Service,
14 Osakidetza/Basque Health Service, Donostia University Hospital, Gipuzkoa. Spain
15 6.	Respiratory and Pleural Diseases Group. Biogipuzkoa Health Research Institute. Thoracic Surgery Service,
16 Osakidetza/Basque Health Service, Donostia University Hospital, Gipuzkoa, Spain
17 7.	Biochemistry Laboratory, Osakidetza/Basque Health Service, Mendaro Hospital, Gipuzkoa, Spain
18 8.	Microbiology Department, Respiratory Infection and Antimicrobial Resistance Group. Osakidetza/Basque Health
19 Service, Biogipuzkoa Health Research Institute, Donostia University Hospital, Gipuzkoa, Spain
20 9.	Biochemistry Laboratory, Osakidetza/Basque Health Service, Donostia University Hospital, Gipuzkoa, Spain
21 10. Oncology area. Biogipuzkoa Health Research Institute.

22

23	Corresponding author: [alberto.garciazamalloa@gmail.com](mailto:alberto.garciazamalloa@gmail.com)

24

## 25 Contribution of each author:

26 -	Conceptualization: Alberto Garcia-Zamalloa, Rafael Arnay, Jorge Taboada.
27 -	Data collection: Alberto Garcia-Zamalloa, Iñaki Salegui, Juan Antonio de Miguel, Nekane
28 Mugica, Borja Aguinagalde, Jon Zabaleta, Begoña Basauri, Marta Alonso, Nekane Azcue,
29 Eva Gil.
30 -	Formal analysis: Rafael Arnay, Iván Castilla-Rodriguez, Javier Mar, Jose Manuel Gonzalez-
31 Cava, Oliver Ibarrondo, Irati Garmendia.
32 -	Funding adquisition: No funding.
33 -	Investigation: Alberto Garcia-Zamalloa, Juan Antonio de Miguel, Borja Aguinagalde, Irati
34 Garmendia.
35 -	Methodology and software: Rafael Arnay, Iván Castilla-Rodriguez, Javier Mar, Jose Manuel
36 Gonzalez-Cava, Oliver Ibarrondo, Irati Garmendia.
37 -	Writing: Alberto Garcia-Zamalloa, Rafael Arnay, Iván Castilla-Rodriguez, Javier Mar.

38

39	Abstract

40

41 Objective.
42 To perform an external validation of a previously reported machine learning (ML) approach for
43 predicting the diagnosis of pleural tuberculosis.
44 Patients and Methods.
45 We defined two cohorts: a Training group, comprising 273 effusions from our prospective study (2013-
46 2020); and a Testing group, from a retrospective analysis of 360 exudative and lymphocytic pleural
47 effusions from 832 consecutive patients in Bajo Deba health district (1996-2012). In training and test
48 groups respectively, 49 and 104 cases were tuberculous, 143 and 92 were malignant, and 81 and
49 164 were diagnosed with “other diseases”; pre-test probabilities of pleural tuberculosis were 4% and
50 12.7%. Variables included were: age, pH, adenosine deaminase, glucose, protein, and lactate
51 dehydrogenase levels, and white cell counts (total and differential) in pleural fluid. We used two ML
52 classifiers: binary (tuberculous and non-tuberculous), and three-class (tuberculous, malignant, and
53 others); and compared them with Bayesian analysis.
54 Results.
55 The best binary classifier yielded a sensitivity of 88%, specificity of 98%, and accuracy of 95%. The
56 best three-class classifier achieved the same accuracy and correctly classified 83% (77/92) of
57 malignant cases. The ML models yielded higher positive predictive values than Bayesian analysis
58 based on ADA > 40 U/l and lymphocyte percentage ≥ 50% (92%).
59 Conclusions.
60 This external validation confirms the good performance of the previously reported ML approach for
61 predicting the diagnosis of pleural tuberculosis based on exudative and lymphocytic pleural effusions,
62 and for discriminating the cases most likely to be malignant. Additionally, ML was more accurate than
63 the Bayesian approach in our study.
64 WORD COUNT: 249

# 65 Introduction

66 Tuberculosis (TB) remains a major global public health problem. In 2024, the World Health
67 Organization estimated that 10.8 million people worldwide had TB (95% UI: 10.1-11.7 million) and
68 1.25 million of them died of this disease through 2023 [1]. Tuberculous pleural effusion (TPE) is the
69 second most prevalent manifestation of extrapulmonary TB, just after tuberculous lymphadenitis [2].
70 Additionally, TPE constitutes a paucibacillary manifestation of tuberculous disease, and the
71 usefulness of various biomarkers in pleural fluid has been extensively analysed. The most promising
72 biomarker is adenosine deaminase (ADA), as the measurement of this enzyme is cheap, easy and
73 available in most laboratories, and it has shown uniformly high diagnostic performance through five
74 consecutive meta-analyses with a mean sensitivity and specificity of 92-93% and 90-92% respectively,
75 for a diagnostic cut-off point of 40 U/l [3–7].
76 The approach for diagnosing incident cases has traditionally been determined by the regional
77 variation in overall TPE prevalence [8]; in relation to this, based on a Bayesian interpretation of its
78 diagnostic accuracy, ADA in pleural fluid is considered optimal as a rule-in test in high TB prevalence
79 settings and as a rule-out test in low prevalence scenarios [9, 10]. More recently, Shaw et al. proposed
80 an ADA level greater than 40 U/L for ruling in TPE diagnosis with a local TB global incidence of over
81 125 cases per 100,000 population, and ADA less than 30 U/L for ruling out TPE in low-incidence
82 countries [11].
83 The progressively wider use of machine learning (ML) tools in medicine is providing researchers with
84 new opportunities to improve diagnostic accuracy by including additional biomarkers. In 2021, our
85 group published a comparison of different ML algorithms for the diagnosis of TPE in a low prevalence
86 scenario (3.8%) based on 230 exudative and lymphocytic pleural effusions [12]. The variables
87 included in the predictive models were age, ADA and results from the routine analysis of pleural fluid
88 including cell counts and biochemical tests. The best ML algorithm (support vector classifier, SVC, a
89 particular type of support vector machine) achieved a sensitivity and specificity of 91% and 98%
90 respectively; furthermore, compared with the Bayesian analysis of ADA level > 40 U/l plus lymphocyte
91 percentage ≥ 50% in pleural fluid, the positive predictive value (PPV) increased from 42.4% to 70.5%
92 in a 5% pre-test probability scenario. The model is freely available as an app (at
93 <https://pleurapp.ispana.es/>) to help other physicians or thoracic surgeons apply this approach when
94 dealing with exudative and lymphocytic pleural effusions. As the presumptive diagnosis of TPE has
95 been traditionally driven by its prevalence, we needed to confirm that the implemented tool maintains
96 its diagnostic properties in other scenarios featuring a higher tuberculous prevalence.
97 The aim of this study was to perform an external validation of the aforementioned model by testing
98 the trained ML algorithm in a higher TPE prevalence setting. In a second step, we compared the
99 diagnostic accuracy of the ML procedure and the classical Bayesian analysis system for TPE in both
100 different clinical scenarios (Bajo Deba 1996-2012 and Gipuzkoa 2013-2022).

101

# 102 Materials and methods

103 We pooled data from two groups of patients from Gipuzkoa (Basque Country, Spain) from January
104 1996 to the present. All the patients had been diagnosed with pleural effusion, undergone diagnostic
105 thoracentesis, and had ADA level measured in the pleural fluid sample obtained. The first group, with
106 which the ML models were tested (Testing group), comprised a retrospective cohort of patients from
107 a specific area (Bajo Deba health district) for whom data were collected from January 1996 to
108 December 2012 (partial results from 1998 to 2008 were published in 2012) [13]. The second group,
109 with which the ML models were trained (Training group), corresponded to patients from the whole
110 region of Gipuzkoa, included in a previously published project regarding the diagnostic accuracy of
111 ML for TPE [12], and for whom data were collected from January 2013 to December 2020. We
112 extended this group with patients prospectively recruited across the region until December 2022. The
113 retrospective project counted with the permission of the local Ethics Committee of the Mendaro
114 Hospital and the approval of the Western Gipuzkoa Clinical Research Commission; in the prospective
115 project all patients gave written informed consent (the parents of a seventeen year old girl signed the
116 unique informed consent for a minor) and the protocol was evaluated and approved by the Clinical
117 Research Ethics Committee of Gipuzkoa (Record number 11/12). Data were accessed for research
118 purposes from September to December 2012 within the retrospective project and from March 2013
119 to December 2022 for the prospective one.

120

121 The mean annual incidence rates of TB in Bajo Deba health district from 1996 to 2012 and in
122 Gipuzkoa from 2013 to 2022 were 43.17 and 12.59 cases per 100,000 population respectively.
123 Although a Tuberculosis Control Programme was not implemented in the Basque Country until 2003
124 [14], data recording has been highly reliable since 1995.
125 The following variables were included: age, pleural fluid ADA level, and pleural fluid routine test
126 results: pH, glucose, protein, and lactate dehydrogenase (LDH) levels, and white blood cell counts
127 (total and differential). It was not possible to include the red blood cell count in the analysis since it
128 had not been routinely assessed in the referral hospital of Bajo Deba health district during the
129 specified period (1996-2012). Further, to ensure consistency with the methodology employed in the
130 2013-2022 Gipuzkoa study, we considered only the first pleural fluid sample from each case. The
131 databases were anonymized and authors did not have access to information that could identify
132 individual participants.
133 Diagnostic criteria for tuberculous, malignant and parapneumonic pleural effusion applied to all the
134 cases from the external validation/Testing group were as follows [15, 16]:
135 •	Confirmed tuberculous pleural effusion: positive culture or Xpert MTB/RIF assay in pleural
136 fluid, pleural tissue or sputum.
137 •	Probable tuberculous pleural effusion: granulomatous inflammation in pleural tissue and/or
138 exudative and lymphocytic pleural effusion with ADA > 40 U/l and complete recovery with
139 antituberculosis treatment.
140 •	Confirmed malignant pleural effusion: malignant cells found in pleural biopsy tissue or pleural
141 fluid.
142 •	Paramalignant pleural effusion: cancer diagnosed de novo, no other cause of pleural effusion
143 identified, absence of malignant cells in pleural fluid and pleural biopsy tissue, and parallel
144 evolution of malignant disease and pleural effusion.

| 145 | - Parapneumonic effusion: alveolar consolidation diagnosed by chest X-ray or computed |
| --- | --- |
| 146 | tomography with ipsilateral pleural effusion, and total recovery of both with antibiotic |
| 147 | treatment. |
| 148 |  |
| 149 | The rest of the cases in this group were diagnosed by well-defined clinical criteria, and all the cases |
| 150 | in the Training group had gold standard quality diagnoses [12]. |
| 151 | Overall, in the Testing group (Bajo Deba 1996-2012), 360 out of 832 episodes of pleural effusion |
| 152 | were exudative and lymphocytic, and TB was diagnosed in 104 out of these 360 cases. Additionally, |
| 153 | 92 cases were found to have a malignant aetiology. Regarding the Training group (Gipuzkoa 2013- |
| 154 | 2022), 273 pleural effusions were exudative and lymphocytic and, of these, 49 were of tuberculous |
| 155 | origin and 143 malignant. |
| 156 | In line with previous studies on pleural diseases since the 1990s, the term “prevalence” was employed |
| 157 | to refer to the number of cases of a specific type of pleural effusion divided by the total number of |
| 158 | pleural effusions studied in a given clinical setting over a known period of time. In this context, |
| 159 | “prevalence” can be considered a synonym of “pre-test probability”. Nonetheless, it should be noted |
| 160 | that the term “pre-test probability” for ML specifically refers to the number of pleural fluid samples with |
| 161 | a given diagnosis divided by the number of pleural fluid samples included in the study, not in the |
| 162 | clinical scenario. Table 1 reports both of these variables; additionally, Supplementary Tables 1 and 2 |
| 163 | list the aetiology and diagnostic criteria met for all the cases included in the study. |
| 164  165 | Table 1: Number of cases in the Training and Testing groups; pre-test probability for 1) local prevalence of |
| 166 | tuberculous pleural effusion and 2) cases included in the machine learning analysis (that is, considering only the |
| 167 | cases with exudative and lymphocytic effusion samples). |

| Group | BAJO DEBA 1996-  2012 (Testing group) | GIPUZKOA 2013-2022  (Training group) |
| --- | --- | --- |
| Cases with thoracentesis, (n) | 832 | 1220 |
| Exudative and lymphocytic pleural effusions included, (n) | 360 | 273 |
| Non-tuberculous cases (based on exudative and lymphocytic  effusions), (n) | 256 | 224 |
| Cases of tuberculosis, (n) | 104 | 49 |

| Local pre-test probability or “prevalence” | 12.5% (104/832) | 4% (49/1220) |
| --- | --- | --- |
| Machine learning pre-test probability or “prevalence” | 28.8% (104/360) | 17.9% (49/273) |
| Malignant cases, (n) | 92 | 143 |
| Other cases, (n) | 164 | 81 |

168

169 Age and pleural fluid ADA level were by far the most relevant variables in our reference study
170 (Training Group), and therefore, we compared the two populations as a function of these two variables
171 and in a categorical way. Results of these comparisons are presented in Table 2.
172 As we can see, “age” and “ADA level” show similar values and behaviour in the two groups. The only
173 statistically significant difference was found in the age of the two populations diagnosed with TPE:
174 they were younger in the external validation or Testing group (Bajo Deba 1996-2012), and this is
175 attributable to the TB incidence in the corresponding period being higher than in the Training group
176 (Gipuzkoa 2013-2022), implying more TB bacilli circulating in the community and in turn a higher
177 proportion of primary infections like cases of TPE in young people [17, 18].

178

179 Table 2: Comparative analysis of categorical variables age and ADA level in the reference (Training) and external
180 validation (Testing) groups (considering *p* < 0.05 indicative of a statistically significant difference).

| Diagnosis | | Tuberculosis | | | Malignancy | | | Others | | |
| --- | --- | --- | --- | --- | --- | --- | --- | --- | --- | --- |
| Patient group | | Bajo Deba  1996-2012 | Gipuzkoa  2013-2022 | *p*  value | Bajo Deba  1996-2012 | Gipuzkoa  2013-2022 | *p*  value | Bajo Deba  1996-2012 | Gipuzkoa  2013-2022 | *p*  value |
| Number of  cases | | 104 | 49 | **-** | 92 | 143 | *-* | 164 | 81 | *-* |
| Age (years) | <30 | 57  (54.81%) | 7 (14.29%) | **<0.001** | 0 (0.00%) | 0 (0.00%) | 0.191 | 3 (1.83%) | 2 (2.47%) | 0.872 |
|  | 30-60 | 22  (21.15%) | 22  (44.90%) |  | 15  (16.30%) | 34 (23.78%) |  | 36 (21.95%) | 19 (23.46%) |  |
|  | ≥60 | 25  (24.04%) | 20  (40.82%) |  | 77  (83.70%) | 109  (76.22%) |  | 125 (76.22%) | 60 (74.07%) |  |
| ADA | <=40  U/l | 8 (7.69%) | 2 (4.08%) | 0.503 | 89  (96.74%) | 133  (93.01%) | 0.258 | 159 (96.95%) | 77 (95.06%) | 0.483 |
|  | >40  U/l | 96  (92.31%) | 47  (95.92%) |  | 3 (3.26%) | 10 (6.99%) |  | 5 (3.05%) | 4 (4.94%) |  |

181

182 Along with this, Figure 1 shows the distribution of “tuberculous” and “others” samples as a function of
183 ADA and age in both Training and Testing groups (recalling that all pleural fluid samples are exudative
184 and lymphocytic, and hence, this distribution is the result of combining the two variables ADA > 40
185 U/l and lymphocyte percentage ≥ 50%: ADA 40 + LP 50). In this figure, there is a horizontal line
186 representing the ADA level = 40 U/l. We can conclude that patients in both Training and Testing
187 groups were evenly distributed, and hence, a comparison between them is valid.

188

189

190 Figure 1: Distribution of “Tuberculosis” and “others” samples in terms of ADA and age in Training (Gipuzkoa
191 2013-2022, left) and Testing (Bajo Deba 1996-2012, right) groups.

192

# 193 Results

194 Two distinct analyses were conducted. The primary analysis employed ML techniques to categorize
195 samples into two groups: tuberculous and non-tuberculous. A secondary analysis utilized the same
196 techniques to further differentiate malignant cases, resulting in three categories: tuberculous,
197 malignant, and "others". During both the training and testing phases, the following variables were
198 used: age, pleural fluid ADA level, and routine parameters derived from pleural fluid analysis
199 (including pH, glucose, protein, and LDH levels, and total and differential white blood cell counts). In
200 this study, the six classifiers employed were the same as those used in the previous study, and are
201 among the most widely used types of ML classifier: multilayer perceptron, logistic regression, SVC,
202 decision tree, K-nearest neighbours, and random forest. As in our previous study, we utilized the
203 Python scikit-learn library to implement these classifiers. For each classifier, we explored a range of
204 parameter values in conjunction with a 5-fold cross-validation approach.

### 205 Binary classification: tuberculosis and others

206 Figure 2 shows the confusion matrices of the cross-validation results for all the ML models in the
207 training set (Gipuzkoa), and the testing results (Bajo Deba) (0: others, 1: tuberculous; predicted
208 values in columns, real values in rows).

209

210

211

212

Figure 2: Confusion matrices in cross-validation (Gipuzkoa, left) and in testing (Bajo Deba, right).

213 We also conducted the same classification using the ADA 40 + LP 50 criterion to classify a sample
214 as tuberculous. Using this criterion, we classified 210 cases of non-tuberculous disease as non-
215 tuberculous (true negatives, TNs), 47 cases of TB as tuberculous (true positives, TPs), 14 cases of
216 non-tuberculous disease as tuberculous (false positives, FPs) and 2 cases of TB as non-tuberculous
217 disease (false negatives, FNs) in the Gipuzkoa dataset. On the other hand, in the Bajo Deba dataset,
218 we obtained 248 TNs, 96 TPs, 8 FPs and 8 FNs.
219 Table 3 lists the metrics of accuracy, sensitivity, specificity, and PPV for each dataset for the different
220 trained ML models and the ADA 40 + LP 50 criterion.

221

222

223 Table 3: Accuracy, sensitivity, specificity, and positive predictive value for each dataset for the trained machine
224 learning models.

| Classifier type | Gipuzkoa dataset | | | | Bajo Deba dataset | | | |
| --- | --- | --- | --- | --- | --- | --- | --- | --- |
|  | Accuracy | Sensitivity | Specificity | PPV | Accuracy | Sensitivity | Specificity | PPV |
| Logistic regression | 0.95 | 0.98 | 0.94 | 0.77 | 0.94 | 0.86 | 0.98 | 0.95 |
| Support vector machine | 0.96 | 0.98 | 0.96 | 0.83 | 0.95 | 0.88 | 0.98 | 0.94 |
| Decision tree | 0.9 | 1.0 | 0.88 | 0.65 | 0.94 | 0.95 | 0.93 | 0.85 |
| K-nearest neighbours | 0.92 | 0.9 | 0.92 | 0.71 | 0.95 | 0.91 | 0.96 | 0.91 |
| Random forest | 0.95 | 0.96 | 0.94 | 0.78 | 0.95 | 0.9 | 0.96 | 0.91 |
| Multilayer perceptron | 0.96 | 0.94 | 0.96 | 0.85 | 0.94 | 0.82 | 0.98 | 0.96 |
| ADA 40+LP 50 | 0.94 | 0.96 | 0.94 | 0.77 | 0.96 | 0.92 | 0.97 | 0.92 |

PPV: positive predictive value; ADA 40 + LP 50: adenosine deaminase > 40 U/l and lymphocyte percentage ≥ 50%

225

### 226 Comparative analysis of ML and Bayesian analysis for estimating positive and negative

227 predictive values as a function of pre-test probability.
228 In Figure 3, the comparison of positive and negative predictive values as a function of pre-test
229 probability is shown for the different trained ML models, as well as for a classification based on the
230 ADA 40 + LP 50 criterion. The dashed vertical lines indicate the ML TPE pre-test probability points
231 for the training (0.18) and testing (0.29) datasets (recall that the corresponding clinical scenario pre-
232 test probabilities or “local prevalence of TPE” are 4% and 12.5% respectively).

233

234

235

236

Figure 3: Positive and negative predictive values as a function of pre-test probability.

237 The estimated PPV area is calculated as a function of the pre-test probability (prevalence) using the
238 sensitivity and specificity of each classifier obtained in the training dataset (Gipuzkoa):
239 (sensitivity * prevalence) / ((sensitivity * prevalence) + (1 - specificity) * (1 - prevalence)).
240 In Table 4, we can compare the real and estimated PPVs obtained for each ML model as well as for
241 a classification based on ADA 40 + LP 50, at the particular pre-test probabilities in the training
242 (Gipuzkoa: 0.18) and testing (Bajo Deba: 0.29) datasets. The real PPVs in Gipuzkoa and Bajo Deba
243 are calculated as the true positives divided by the sum of the true positives and false positives,
244 obtained in each dataset:
245 TP / (TP + FP)
246 It can be observed that the PPV from the ML models exceeds that based on the ADA 40 + LP 50
247 criterion in several cases, both for the Gipuzkoa and Bajo Deba datasets. Additionally, the estimated
248 PPVs underestimate the real values obtained in the Bajo Deba dataset for all ML models as well as
249 for the ADA 40 + LP 50-based classifier.

250

251

252 Table 4: Real and estimated positive predictive values in the training and testing datasets.

| Classifier type | Gipuzkoa dataset | | Bajo Deba dataset | |
| --- | --- | --- | --- | --- |
|  | Estimated | Real | Estimated | Real |
| Logistic regression | 0.78 | 0.77 | 0.87 | 0.95 |
| Support vector machine | 0.84 | 0.83 | 0.91 | 0.94 |
| Decision tree | 0.64 | 0.65 | 0.77 | 0.85 |
| K-nearest neighbours | 0.71 | 0.71 | 0.82 | 0.91 |
| Random forest | 0.77 | 0.78 | 0.87 | 0.91 |
| Multilayer perceptron | 0.83 | 0.85 | 0.91 | 0.96 |
| ADA 40+LP50 | 0.77 | 0.77 | 0.86 | 0.92 |

ADA 40 + LP 50: adenosine deaminase > 40 U/l and lymphocyte percentage ≥ 50%

### 253 Three-class classification: tuberculous, malignant and others

254 Figure 4 shows the confusion matrices of the classification results in Gipuzkoa (left) and Bajo Deba
255 (right), respectively (0: tuberculous, 1: malignant, and 2: others; predicted values in columns, real
256 values in rows).

257

258

259 Figure 4: Confusion matrices for each classifier in Gipuzkoa (left) and Bajo Deba (right) (0: tuberculous, 1:
260 malignant, 2: others; predicted values in columns, real values in rows).

261

262

# 263 Discussion

264 In this study, we have performed an external validation of our results from a prospective study that
265 assessed the usefulness of ML for predicting the diagnosis of TPE, going beyond the ADA 40 + LP
266 50 criterion, in a low prevalence setting and through the period 2013-2022[12]. We decided to conduct
267 the study in a different prevalence scenario and we found some additional interesting behaviours of
268 the ML classifiers in this context.
269 TPE constitutes a type 4 hypersensitivity reaction to mycobacterial antigens, and it is not certain
270 whether this immunogenic response needs live mycobacteria. There is a well-proven initial invasion
271 of polymorphonuclear leukocytes in the pleural fluid, followed by macrophages, and finally, almost all
272 TPE fluids become lymphocytic[19, 20]. An ideal diagnostic tool to confirm TPE should be able to
273 detect *Mycobacterium tuberculosis* or any of its specific subunits or components directly, and
274 optimally in pleural fluid through diagnostic thoracentesis; but due to its paucibacillary nature, current
275 microbiological and nucleic acid amplification tests show low sensitivity: <5% with acid-fast bacilli
276 smear in pleural fluid, 30-50% with mycobacterial culture and 21-47% with a nucleic acid amplification
277 test compared to a composite reference standard[21]. Pleural biopsy (closed or guided by video-
278 thoracoscopy) is more sensitive but also more invasive, and hence, it is not suitable in all clinical
279 scenarios; further, *Mycobacterium tuberculosis* grows in pleural tissue culture in 75% of cases, the
280 others being diagnosed based on the histological finding of granuloma in pleural tissue, which shows
281 a specificity of 95% but gives no information regarding microbiological characteristics or drug
282 resistance[22].
283 Due to all the above, great efforts have been made over recent decades to obtain a surrogate
284 diagnostic tool for TPE, pleural fluid biomarkers being the most extensively explored. They provide a
285 probability for ruling in and/or ruling out the disease, and among them, ADA is the most cost-effective,
286 it is standardized and it has a universally accepted diagnostic cut-off value (40 U/l) [12]. Its
287 combination with lymphocyte percentage increases ADA’s specificity [23], as we also confirmed in
288 our first study from 1998 to 2008 [13]. In our second study [12], we included both variables in an ML
289 approach. This third study is an external validation of the latter, and we performed it in a higher TPE
290 prevalence scenario.
291 Using the ADA 40 + LP 50 criterion, specificity decreases when moving from a high- to a low-
292 incidence scenario. This is because, proportionally, there are more malignant samples with ADA >
293 40 U/l in low-incidence scenarios, which increases the number of false positives (8 classified
294 erroneously as tuberculous out of a total of 256 non-tuberculous samples in the case of Bajo Deba,
295 compared to 14 classified erroneously as tuberculous out of a total of 224 non-tuberculous samples
296 in the case of Gipuzkoa).
297 Nonetheless, some ML models show a less marked decrease in specificity when transitioning from
298 the high- to the low-incidence scenario. For example, the SVC achieved sensitivities and specificities
299 of 98% and 96% in Gipuzkoa and 88% and 98% in Bajo Deba, while the logistic regression classifier
300 provided sensitivities and specificities, respectively, of 98% and 94% in Gipuzkoa and 86% and 98%
301 in Bajo Deba. This is because these models rely not only on the ADA 40 + LP 50 criterion but also a
302 combination of other features, each with its relative influence, ADA and age being the most important
303 ones.
304 Both the ADA 40 + LP 50 criterion and the ML models provide lower sensitivity in Gipuzkoa than in
305 Bajo Deba health district. This can be explained by two factors. Firstly, there were quite a few cases
306 of TB in Bajo Deba with ADA below 40 U/l (in the first pleural fluid sample), while there are virtually
307 no such cases in Gipuzkoa, causing both the ML models and the ADA > 40 criterion to fail to
308 generalize correctly in the Bajo Deba dataset. Secondly, and this affects only the ML models, the
309 patterns of ADA and age, the two most discriminatory variables, are not the same in the Gipuzkoa
310 and Bajo Deba datasets. Figure 5 shows the tuberculous, malignant, and other cases as a function
311 of ADA and age in Gipuzkoa (left) and in Bajo Deba (right). False negatives produced by the SVC in
312 the Bajo Deba dataset have also been marked with red crosses. As can be seen, there was much
313 more TB circulating in the young population in the Bajo Deba dataset (right) than in Gipuzkoa (left),
314 and although the ML models have not seen this pattern in the training set, they are capable of
315 generalizing well, since there were no non-tuberculous samples in those ranges of ADA and age in
316 the Gipuzkoa dataset either. Nonetheless, there are ranges of ADA slightly above 40 U/l and ages
317 around 80 years, where most samples in the Gipuzkoa dataset are classified as “malignant” or “other”,
318 while in the Bajo Deba dataset, there are tuberculous samples, and hence, most of the trained ML
319 models fail to generalize well.

320

321

322 Figure 5: Tuberculous, malignant, and other cases as a function of adenosine deaminase level and age in
323 Gipuzkoa (left) and in Bajo Deba (right). False negatives produced by the support vector classifier in the Bajo
324 Deba dataset are marked with red crosses.

325

326 Comparing ML and Bayesian analysis, it can be observed that the PPV of the ML models exceeds
327 that of ADA 40 + LP 50 in several cases, for both the Gipuzkoa and Bajo Deba datasets. Additionally,
328 the estimated PPVs underestimate the real values obtained in the Bajo Deba dataset for all ML
329 models as well as for the ADA 40 + LP 50-based classifier (Figure 3 and Table 4). This indicates that
330 the “tuberculous” and “others” classes are more readily separable in the Bajo Deba than in the
331 Gipuzkoa dataset. This is primarily attributable to the fact that there are fewer malignant cases with
332 high ADA in Bajo Deba than in Gipuzkoa. Consequently, the projected PPV for a classifier with a
333 sensitivity and specificity obtained in Gipuzkoa falls short of the real PPV obtained in Bajo Deba for
334 that classifier.
335 Concerning the results of the three-class classification (tuberculous/malignant/others), as can be
336 observed, practically all three-class classifiers provide sensitivity/specificity in the classification of
337 tuberculous cases against the others similar to that of the two-class classifiers. For example, the
338 Random Forest classifier correctly detects 47 cases of TPE out of a total of 49 in Gipuzkoa and 92
339 out of a total of 104 in Bajo Deba, but at the same time, it is capable of correctly classifying 118
340 malignant cases out of a total of 143 malignant cases in Gipuzkoa, as well as correctly classifying 74
341 malignant cases out of a total of 92 malignant cases in Bajo Deba (Figure 4).
342 This study has some limitations. First, the analysis of the testing dataset is retrospective, it has been
343 performed in a single centre and the quality of diagnosis is lower than in the training dataset. Second,
344 the incidence rate of TB is higher in the testing group, but at the same time, this has provided an
345 opportunity to analyse the behaviour of the ML classifiers in different prevalence scenarios of a
346 transmissible disease. Moreover, the number of cases included in the study, the homogeneity of the
347 groups and the comparable behaviour of the two most important variables are positive features,
348 enhancing the validity of our study’s findings.

349

350	Conclusions

| 351 | With this external validation of our previously reported model, we confirm that an ML approach |
| --- | --- |
| 352 | combining ADA with age and routine pleural fluid parameters is suitable for predicting the diagnosis |
| 353 | of pleural TB in any prevalence scenario, and secondarily, for discriminating the cases most likely to |
| 354 | be malignant amongst exudative and lymphocytic non-tuberculous effusions. Additionally, we |
| 355 | conclude that ML is more accurate than Bayesian analysis in different prevalence scenarios, although |
| 356 | the epidemiological behaviour of transmissible diseases (e.g., the relationship between incidence rate |
| 357  358  359 | and patient age) is a challenge to be tackled in developing ML approaches. |
| 360  361  362  363 | References |
| 364 | 1 Global tuberculosis report 2024 [Internet]. Geneva: World Health Organization; 2024. Available from: |
| 365 | https://[www.who.int/teams/global-tuberculosis-programme/tb-reports/global-tuberculosis-report-2024/tb-](http://www.who.int/teams/global-tuberculosis-programme/tb-reports/global-tuberculosis-report-2024/tb-) |
| 366 | disease-burden |
| 367 | 2 Cohen LA, Light RW. Tuberculous Pleural Effusion. *Turk Thorac J* 2015; 16: 1–9. |
| 368 | 3 Palma RM, Bielsa S, Esquerda A, *et al.* Diagnostic Accuracy of Pleural Fluid Adenosine Deaminase for |
| 369 | Diagnosing Tuberculosis. Meta-analysis of Spanish Studies. *Arch Bronconeumol (Engl Ed)* 2019; 55: 23–30. |
| 370 | 4 Greco S, Girardi E, Masciangelo R, *et al.* Adenosine deaminase and interferon gamma measurements for the |
| 371 | diagnosis of tuberculous pleurisy: a meta-analysis. *Int J Tuberc Lung Dis* 2003; 7: 777–786. |
| 372 | 5 Liang Q-L, Shi H-Z, Wang K, *et al.* Diagnostic accuracy of adenosine deaminase in tuberculous pleurisy: a |
| 373 | meta-analysis. *Respir Med* 2008; 102: 744–754. |
| 374 | 6Aggarwal AN, Agarwal R, Sehgal IS, *et al.* Adenosine deaminase for diagnosis of tuberculous pleural effusion: |
| 375 | A systematic review and meta-analysis. *PLoS One* 2019; 14: e0213728. |
| 376 | 7 Morisson P, Neves DD. Evaluation of adenosine deaminase in the diagnosis of pleural tuberculosis: a |
| 377 | Brazilian meta-analysis. *J Bras Pneumol* 2008; 34: 217–224. |
| 378 | 8 Mummadi SR, Stoller JK, Lopez R, *et al.* Epidemiology of Adult Pleural Disease in the United States. *Chest* |
| 379 | 2021; 160: 1534–1551. |
| 380 | 9 Porcel JM. Biomarkers in the diagnosis of pleural diseases: a 2018 update. *Ther Adv Respir Dis* 2018; 12: |
| 381 | 175346661880866. doi: 10.1177/1753466618808660. |
| 382 | 10 Antonangelo L, Faria CS, Sales RK. Tuberculous pleural effusion: diagnosis & management. *Expert Rev* |
| 383 | *Respir Med* 2019; 13: 747–759. |

384 11 Shaw JA, Ahmed L, Koegelenberg CFN. Effusions related to TB. In: Maskell NA, Laursen CB, Lee YCG,
385 et al., eds. *Pleural Disease* [Internet] Sheffield, United Kingdom: European Respiratory Society; 2020 [cited
386 2024	May	22].	p.	172–192.	Available	from:
387 [http://erspublications.com/lookup/doi/10.1183/2312508X.10023819.](http://erspublications.com/lookup/doi/10.1183/2312508X.10023819)
388 12 Garcia-Zamalloa A, Vicente D, Arnay R, *et al.* Diagnostic accuracy of adenosine deaminase for pleural
389 tuberculosis in a low prevalence setting: A machine learning approach within a 7-year prospective multi-
390 center study. Quinn F, ed. *PLoS ONE* 2021; 16: e0259203.
391 13 Garcia-Zamalloa A, Taboada-Gomez J. Diagnostic Accuracy of Adenosine Deaminase and Lymphocyte
392 Proportion in Pleural Fluid for Tuberculous Pleurisy in Different Prevalence Scenarios. Caylà JA, ed. *PLoS*
393 *ONE* 2012; 7: e38729.
394 14	Tuberculosis	Prevention	and	Control	Program.	Workgroup.	Consensus	document.;	2001.
395 https://[www.ogasun.ejgv.euskadi.eus/r51-](http://www.ogasun.ejgv.euskadi.eus/r51-)
396 catpub/es/k75aWebPublicacionesWar/k75aObtenerPublicacionDigitalServlet?R01HNoPortal=true&N_LIB
397 R=051622&N_EDIC=0001&C_IDIOM=es&FORMATO=.pdf.
398 15 Roberts ME, Rahman NM, Maskell NA, *et al.* British Thoracic Society Guideline for pleural disease. *Thorax*

399	2023; 78: 1143–1156.

400 16 Herrera Lara S, Fernández-Fabrellas E, Juan Samper G, *et al.* Predicting Malignant and Paramalignant
401 Pleural Effusions by Combining Clinical, Radiological and Pleural Fluid Analytical Parameters. *Lung* 2017;
402 195: 653–660.
403 17 Valdés L, Ferreiro L, Cruz-Ferro E, *et al.* Recent epidemiological trends in tuberculous pleural effusion in
404 Galicia, Spain. *European Journal of Internal Medicine* 2012; 23: 727–732.
405 18 Garcia Zamalloa A, Taboada Gómez J, Arrospide Elgerresta A, *et al.* Pleural tuberculosis in young people
406 as an indicator of general tuberculosis rate. *Eur Respir J* 2014; 44: P2689.
407 19 Vorster MJ, Allwood BW, Diacon AH, *et al.* Tuberculous pleural effusions: advances and controversies. *J*

408	*Thorac Dis* 2015; 7: 981–991.

409 20 Sahn SA, Huggins JT, San José ME, *et al.* Can tuberculous pleural effusions be diagnosed by pleural fluid
410 analysis alone? *Int J Tuberc Lung Dis* 2013; 17: 787–793.
411 21 Chan KKP, Lee YCG. Tuberculous pleuritis: clinical presentations and diagnostic challenges. *Current*
412 *Opinion in Pulmonary Medicine* 2024; 30: 210–216.
413 22 Porcel JM. Advances in the diagnosis of tuberculous pleuritis. *Ann Transl Med* 2016; 4: 282.
414 23 Burgess LJ, Maritz FJ, Le Roux I, *et al.* Combined use of pleural adenosine deaminase with
415 lymphocyte/neutrophil ratio. Increased specificity for the diagnosis of tuberculous pleuritis. *Chest* 1996; 109:
416 414–419.

417

Figure	[Click here to access/download;Figure;Figure 1.docx](https://www2.cloud.editorialmanager.com/pone/download.aspx?id=39449768&guid=2acad878-c5e5-47b6-ac88-064c58ba467f&scheme=1)[<https://www2.cloud.editorialmanager.com/pone/download.aspx?id=39449768&guid=2acad878-c5e5-47b6-ac88-064c58ba467f&scheme=1>](https://www2.cloud.editorialmanager.com/pone/download.aspx?id=39449768&guid=2acad878-c5e5-47b6-ac88-064c58ba467f&scheme=1)

Figure	[Click here to access/download;Figure;Figure 2.docx](https://www2.cloud.editorialmanager.com/pone/download.aspx?id=39449769&guid=e6a499f6-d87c-4908-95b9-5b01b3555ae1&scheme=1)[<https://www2.cloud.editorialmanager.com/pone/download.aspx?id=39449769&guid=e6a499f6-d87c-4908-95b9-5b01b3555ae1&scheme=1>](https://www2.cloud.editorialmanager.com/pone/download.aspx?id=39449769&guid=e6a499f6-d87c-4908-95b9-5b01b3555ae1&scheme=1)

Figure	[Click here to access/download;Figure;Figure 3.docx](https://www2.cloud.editorialmanager.com/pone/download.aspx?id=39449770&guid=4a4a2f6e-ab48-4885-b20d-98bebb6c6836&scheme=1)[<https://www2.cloud.editorialmanager.com/pone/download.aspx?id=39449770&guid=4a4a2f6e-ab48-4885-b20d-98bebb6c6836&scheme=1>](https://www2.cloud.editorialmanager.com/pone/download.aspx?id=39449770&guid=4a4a2f6e-ab48-4885-b20d-98bebb6c6836&scheme=1)

Figure	[Click here to access/download;Figure;Figure 4.docx](https://www2.cloud.editorialmanager.com/pone/download.aspx?id=39449776&guid=099309e5-fdcf-4a96-b0c2-ca6adeefab34&scheme=1)[<https://www2.cloud.editorialmanager.com/pone/download.aspx?id=39449776&guid=099309e5-fdcf-4a96-b0c2-ca6adeefab34&scheme=1>](https://www2.cloud.editorialmanager.com/pone/download.aspx?id=39449776&guid=099309e5-fdcf-4a96-b0c2-ca6adeefab34&scheme=1)

Figure	[Click here to access/download;Figure;Figure 5.docx](https://www2.cloud.editorialmanager.com/pone/download.aspx?id=39449778&guid=10944665-9733-45e2-b29e-cf78af0794df&scheme=1)[<https://www2.cloud.editorialmanager.com/pone/download.aspx?id=39449778&guid=10944665-9733-45e2-b29e-cf78af0794df&scheme=1>](https://www2.cloud.editorialmanager.com/pone/download.aspx?id=39449778&guid=10944665-9733-45e2-b29e-cf78af0794df&scheme=1)

Supporting Information

[Click here to access/download](https://www2.cloud.editorialmanager.com/pone/download.aspx?id=39449783&guid=af34bb20-fd49-4c1d-81ce-93767fd10bef&scheme=1)

[**Supporting Information** 20250221_SUPPLEMENTARY_DATA_EXTERNAL_VAL](https://www2.cloud.editorialmanager.com/pone/download.aspx?id=39449783&guid=af34bb20-fd49-4c1d-81ce-93767fd10bef&scheme=1)

[IDATION.docx](https://www2.cloud.editorialmanager.com/pone/download.aspx?id=39449783&guid=af34bb20-fd49-4c1d-81ce-93767fd10bef&scheme=1)
